# Genome-wide identification of coding and non-coding conserved sequence tags in human and mouse genomes

**DOI:** 10.1186/1471-2164-9-277

**Published:** 2008-06-11

**Authors:** Flavio Mignone, Anna Anselmo, Giacinto Donvito, Giorgio P Maggi, Giorgio Grillo, Graziano Pesole

**Affiliations:** 1 Department of Structural Chemistry and Inorganic Stereochemistry, School of Pharmacy, University of Milan, Italy; 2 Department of Biomolecular Sciences and Biotechnology, University of Milan, Italy; 3 National Institute of Nuclear Physics, Bari, Italy; 4 Istituto Tecnologie Biomediche, Consiglio Nazionale delle Ricerche, Bari, Italy; 5 Dipartimento di Biochimica e Biologia Molecolare, University of Bari, Italy

## Abstract

**Background:**

The accurate detection of genes and the identification of functional regions is still an open issue in the annotation of genomic sequences. This problem affects new genomes but also those of very well studied organisms such as human and mouse where, despite the great efforts, the inventory of genes and regulatory regions is far from complete. Comparative genomics is an effective approach to address this problem. Unfortunately it is limited by the computational requirements needed to perform genome-wide comparisons and by the problem of discriminating between conserved coding and non-coding sequences. This discrimination is often based (thus dependent) on the availability of annotated proteins.

**Results:**

In this paper we present the results of a comprehensive comparison of human and mouse genomes performed with a new high throughput grid-based system which allows the rapid detection of conserved sequences and accurate assessment of their coding potential. By detecting clusters of coding conserved sequences the system is also suitable to accurately identify potential gene loci.

Following this analysis we created a collection of human-mouse conserved sequence tags and carefully compared our results to reliable annotations in order to benchmark the reliability of our classifications. Strikingly we were able to detect several potential gene loci supported by EST sequences but not corresponding to as yet annotated genes.

**Conclusion:**

Here we present a new system which allows comprehensive comparison of genomes to detect conserved coding and non-coding sequences and the identification of potential gene loci. Our system does not require the availability of any annotated sequence thus is suitable for the analysis of new or poorly annotated genomes.

## Background

One of the main challenges of post-genomic era is the accurate annotation of genes and the improvement of our knowledge of mechanisms of gene expression through the identification of cis-acting non-coding regulatory regions. Comparative genomics has been one of the most successful approaches used to address this task. Indeed it is well known that sequences with functional activity – such as coding sequences or regulatory regions – are subject to selective pressures that prevent the fixation of mutations and conserve sequences during evolution.

Conserved non-coding sequences have been shown to act as tissue specific enhancers of gene expression [1] and in particular of genes involved in control of development [2]. Evolutionary conserved sequences have also been successfully used for the identification of new genes [3].

Given the great interest in this area of research and thanks to the availability of the almost complete genome sequences of many organisms, several tools to identify and collect conserved sequences have been proposed [4,5].

The identification of a conserved sequence is only the first step in the identification of functional elements that requires further information, the most obvious being the assessment of its coding potential, i.e. to assess if the conserved sequence is likely to be part of a coding region. Discriminating between coding and non coding conserved sequences is of great importance as the discovery of novel coding sequences may help the detection of unannotated genes or coding exons and the identification of splice variants. Conversely, the study of non-coding conserved sequences may lead to the identification of regions that may have regulatory activity both at DNA or mRNA level by affecting transcription or translation thus modulating gene expression.

The usual approach is to classify conserved sequences as coding or noncoding by comparison with annotated protein sequences: if a conserved sequence is not supported by (does not align to) a known protein it is labelled as non-coding.

This approach makes classification heavily dependent on the quality of the annotation of the genomes under analysis and it is obviously less applicable to new – poorly annotated – genomes.

We previously developed CSTminer, a tool that does not suffer from these limitations as it identifies conserved sequences (Conserved Sequence Tags – CSTs) and classifies them as coding or non-coding by evaluating the presence of evolutionary dynamics specific of coding sequences [6,7].

We recently applied CSTminer to an extensive analysis of human chromosomes 15, 21 and 22 and corresponding mouse syntenic regions [8]. We identified more than 37,000 CSTs. 9,500 of these were labelled as coding and were used to benchmark a novel methodology – based on the identification of clusters of coding CSTs – to detect genomic regions which are likely to contain genes (see [8] for details). One striking result of the work was that, despite the large efforts made towards the annotation of human genome, we were able to identify 25 loci potentially containing unannotated genes using a relatively simple comparative approach. Interestingly 11 of the 25 predicted genes were confirmed by updated genome annotation at the time of publication – confirming the reliability of our approach.

The computational problem for the comprehensive comparison of two genomes of the size of human and mouse is not trivial. Indeed, although CSTminer is fairly fast, it is limited by the alignment step which implements a blast like algorithm which is not suitable to compare very long sequences.

In this paper we propose a highly parallelized system to perform a complete comparison of large genomes. This system also allows the submission of precomputed genomic alignments (such as blastz) to further improve the speed of the analysis.

A preliminary study of sequences conserved between human and mouse genomes allowed the identification of several clusters of coding CSTs that do not correspond to any annotated genes and the creation of a collection of non-coding CSTs possibly endowed of some functional activity.

## Results

The first operation performed by the CSTminer algorithm is the identification of high scoring segment pairs (HSPs) through a Blast-like sequence comparison. A solution to the computational problem associated with whole genome comparisons is to split long sequences into smaller fragments (in order to limit the number of CSTs interrupted at the boundaries of the fragments, these cannot be too short). We empirically established a length of 100 Kbp (with an overlap of 1 Kbp) as a good compromise (data not shown). Given the size of human (~3 Gbp) and mouse (~2.6 Gbp) genomes, an exhaustive comparison between all human and mouse 100 Kbp sequences would require nearly 800 M comparisons. Considering an average computation time of 2 sec for each comparison the whole analysis would require many years of computation on a single CPU. We took advantage of the high level of parallelization offered by grid technology and developed a system suitable to perform all comparisons in a "reasonable" time.

The number of tasks to be performed is very large and in a distributed environment there are many reasons that individual jobs may fail (Worker Node problems, site configuration, problems due to middleware failures, etc). Accurate job management is therefore essential and to this end a fully automated procedure based on mysql DBMS was developed to launch and monitor jobs, re-run failed jobs and to collect results of the analysis.

### Genome-wide detection of human-mouse Conserved Sequence Tags (CSTs)

Following CSTminer analysis we obtained a redundant collection (see below) of nearly 1,500,000 CSTs with an average length of 190 nt as summarized in Table 1 where total number of CSTs, average and maximum length is reported for each chromosome.

**Table 1:** CST distribution among human (A) and mouse (B) chromosomes.

**A**			
**Chromosome**	**Tot**	**AvLen**	**MaxLen**

**chr1**	138165 (66669)	198.01 (187.9948)	4621 (4621)
**chr2**	117207 (64475)	194.56 (196.3639)	17543 (17543)
**chr3**	92770 (51821)	192.64 (191.7276)	3378 (6460)
**chr4**	69640 (28421)	195.49 (190.4989)	4347 (4228)
**chr5**	80407 (37576)	207.3 (195.852)	7219 (7219)
**chr6**	85924 (37799)	203.53 (186.8935)	4177 (4177)
**chr7**	70839 (40048)	189.56 (188.1825)	3829 (3237)
**chr8**	56955 (31423)	188.25 (190.3644)	5600 (5600)
**chr9**	62015 (31661)	186.98 (192.5231)	4403 (4403)
**chr10**	61229 (34765)	193.75 (188.0005)	2997 (3918)
**chr11**	98006 (38038)	167.79 (191.4565)	6512 (4968)
**chr12**	73657 (34444)	180.06 (177.3208)	2680 (2680)
**chr13**	38619 (19341)	194.75 (200.7517)	10176 (10176)
**chr14**	53848 (25668)	191.36 (194.2269)	3854 (3854)
**chr15**	49142 (25707)	201.89 (191.1299)	2600 (2600)
**chr16**	41166 (21161)	185.79 (191.0755)	4020 (4020)
**chr17**	61341 (29899)	172.99 (179.4735)	3179 (3179)
**chr18**	27896 (17053)	198.68 (196.9212)	3719 (3719)
**chr19**	56808 (17624)	144.18 (163.8533)	3738 (3738)
**chr20**	29061 (16007)	195.18 (184.8446)	2862 (2862)
**chr21**	13165 (6688)	174.33 (178.0885)	3935 (3935)
**chr22**	18378 (9414)	180.52 (165.1375)	4710 (4710)
**chrX**	68893 (38838)	200.7 (196.3681)	4882 (4882)
**chrY**	6192 (1786)	171.74 (147.0274)	2087 (3180)

**B**			

**Chromosome**	**Tot**	**AvLen**	**MaxLen**

**chr1**	96565	193.02	3539
**chr2**	123127	180.58	17545
**chr3**	80185	198.2	4615
**chr4**	82789	194.01	4388
**chr5**	76557	184.15	3969
**chr6**	83801	188.59	3251
**chr7**	93077	176.52	6521
**chr8**	63198	194.55	4222
**chr9**	80909	187.01	4222
**chr10**	66720	183.21	4168
**chr11**	97295	176.61	3272
**chr12**	65166	192.03	3857
**chr13**	64693	198.31	3268
**chr14**	59365	200.92	10179
**chr15**	57968	180.62	5600
**chr16**	48185	192.52	3950
**chr17**	54366	185.28	3999
**chr18**	48736	215.77	7206
**chr19**	42161	188.74	5381
**chrX**	85471	199.71	4877
**chrY**	989	145,88	1193

The total CST length (Tot), the average (AvLen) and the maximum length (MaxLen) is reported. Numbers in brackets refer to the non-redundant CST dataset. Minimum length has been limited to 60 nt (see text for details).

The minimum CST length has been limited to 60 nt as shorter sequences would not allow a reliable computation of the coding potential score (see below). Chromosomes 2 and 13 show some uncommonly long CSTs (17,543 and 10,176 nt respectively). These two CSTs are characterized by a high coding potential and correspond to conserved sequences of the long coding exons of TTN and SACS genes.

**Figure 1:**
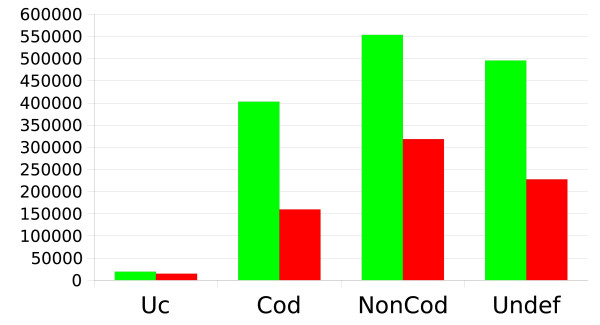
**CST Coding Potential distribution**. Distribution of CSTs among different coding classes assigned by CSTminer. Uc: ultraconserved sequences (identity >95%), Cod: coding CSTs, NonCod: non-coding CSTs, Undef: CSTs with undefined coding potential. Redundant CST dataset is shown with green bars, non-redundant set with red bars. See text for details.

CSTminer assesses the coding potential of CSTs by computing a Coding Potential Score (CPS) based on the evolutionary dynamics observed between the two sequences. Once fixed threshold values for CPS derived from benchmark sets of coding and non-coding sequences a specific label can be assigned to each CST [7]. More than 400,000 CSTs were labelled as coding, 550,000 CSTs were labelled as non-coding, 500,000 CSTs remained undefined as the CPS fell in a twilight zone between the coding and the non-coding thresholds. Finally nearly 20,000 CSTs displayed more than 95% of identity and were labelled as ultra-conserved (Figure 1). Given the low divergence, it is not possible to compute a reliable CPS for these CSTs.

We observed that CSTs labelled as undefined often overlap both coding and non-coding regions corresponding to CDS or to UTR or intron sequences, respectively (data not shown). The global coding potential score assigned by CSTminer is influenced by both subregions and does not allow a clear classification of the sequence.

### Human mouse conservation

**Figure 2:**
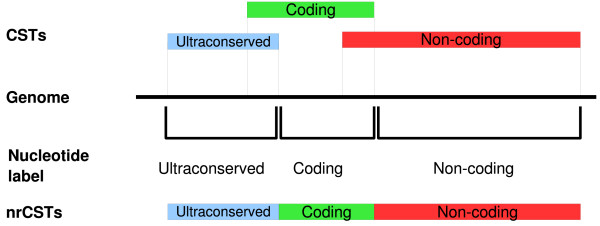
**Non redundant CSTs dataset generation**. Each nucleotide of human and mouse genomes has been labelled as ultraconserved, coding, non-coding or undefined if contained in a CST. Nucleotides part of overlapping CSTs were labelled according to the ranking ultraconserved > coding > non-coding > undefined. Stretches of at least 60 consecutive nucleotides with the same label were merged into a non-redundant CST (nrCST).

In order to accurately evaluate whole human-mouse genome conservation it was necessary to consider that CSTs may overlap one another as the same region of a genome may share similarity with more than one region of the other genome under analysis (mainly due to segmental duplications or paralogous sequences). We projected each CST onto the genome and labelled each human and mouse nucleotide according to the CST label. If the same nucleotide was contained in more than one CST it was labelled according to the ranking: ultraconserved > coding > noncoding > undefined, as depicted in Figure 2. In this way we obtained a non-redundant set of CSTs assigning to each conserved nucleotide an unambiguous annotation.

We then obtained a cleaned set of more than 720,000 nonredundant CSTs (nrCSTs) distributed across human chromosomes (Table 1 numbers in brackets) and labelled as summarized in Figure 1.

**Figure 3:**
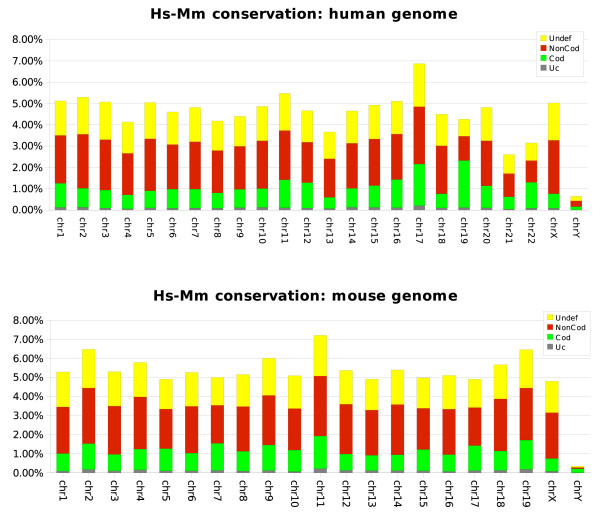
**Human and mouse chromosome conservation**. Chromosome conservation evaluated on human (**A**) and mouse (**B**) genomes. For each chromosome the frequency of conserved nucleotides – evaluated on nrCSTs – is reported. As described in legend each colour corresponds to a coding potential classification. Undef: undefined, NonCod: non-coding, Cod: coding, Uc: ultraconserved sequences (identity>95%).

We evaluated nucleotide conservation of each human and mouse chromosome as shown in Figure 3. Consistent with previous data [9] we observed that average conservation of human and mouse genomes (evaluated as the frequency of conserved nucleotides part of nrCSTs) is around 5%.

Previous observations showed that the majority of genes on human chromosome 17 have their homologues on mouse chromosome 11. Interestingly our data show that these chromosomes are the most conserved, with 6.9% and 7.2% of conserved nucleotides respectively.

Conversely, both human and mouse chromosomes Y are poorly conserved (about 0.5%). This observation could be explained with the degeneration process faced by Y chromosome. Moreover they are unusually rich of repetitive elements that we masked before running our analysis.

Only 0.94% of human genome is labelled as coding from our comparison with mouse genome.

### CSTs and annotated mRNAs

**Figure 4:**
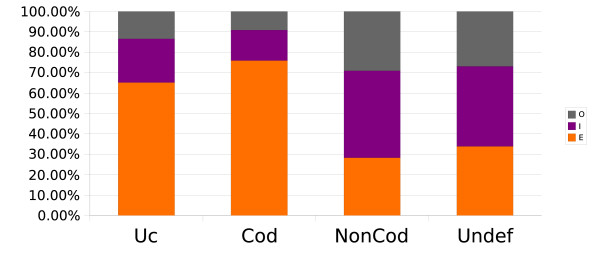
**CSTs comparison to human and mouse mRNAs**. CSTs have been compared to human and mouse annotated mRNAs and classified as Exonic (E) if overlapping with an exon in either human or mouse genomes. Remaining CSTs have been labelled as Intronic (I) if overlapping with an intron, or Intergenic (O) if not overlapping to any annotated gene in both human and mouse genomes. The distribution among different classes is reported for ultraconserved (Uc), coding, non-coding (NonCod) and undefined (Undef) CSTs.

Each CST is characterized by absolute coordinates on both human and mouse genomes and this allows a comparison with mRNA annotations (extracted by UCSC database). We then classified CSTs as exonic, intronic or intergenic on the basis of their overlap with the available mRNAs annotation. Figure 4 shows that 65% of ultraconserved CSTs and 76% of coding CSTs overlap with known exons, 21% and 15% respectively map within introns while the remaining 13% and 9% map outside of annotated genes. Noncoding CSTs – as expected – show a different pattern of localization: 43% and 29% map within intronic or intergenic regions while 28% overlap with annotated exons, likely corresponding to conserved tracts of untranslated regions (UTRs). A similar pattern is also shown by undefined sequences.

### Coding CST-Clustering

To identify regions with an high density of coding conserved sequences – likely gene loci – we applied an improved version of a clustering procedure previously described [8] (see also Material and Methods) and detected 25,296 clusters containing 141,001 CSTs.

By comparing the genomic coordinates of the clusters of coding CSTs with those of annotated mouse and human mRNAs we observed that 22,360 clusters (97.6%) overlapped known mRNAs in at least one organism. Noticeably 15,275 clusters were fully confirmed by human sequences (each CST of the cluster was confirmed by the overlap with an mRNA derived exon) and 15,194 clusters were fully confirmed by mouse sequences (Table 2). These observations underline the reliability of our simple approach to localize gene loci on unannotated genomes and strongly support the idea that clusters not overlapping with known sequences are likely to represent unannotated gene loci.

**Table 2:** Clusters of coding CSTs have been identified as described in text and have been compared to annotated RefSeq mRNAs.

**RefSeq**	
**Tot Clusters**	25296
**Hs confirmed Clusters (full confirmed)**	22360 (15275)
**Mm confirmed Clusters (full confirmed)**	22324 (15194)
**Tot confirmed Clusters**	25297

**ESTs**	

**Tot Clusters unconfirmed by RefSeq**	668
**Hs confirmed Clusters (full confirmed)**	551 (432)

Clusters not corresponding to any annotated mRNA sequence have been compared to ESTs to find evidence of their expression.

Moreover, given that our approach does not require the previous availability of annotated features, it seems reasonable to think that it could prove to be a powerful tool in the annotation of genomes lacking a well curated gene annotation.

As 668 clusters were confirmed neither by human nor by mouse mRNAs, we compared their chromosome coordinates to those of human ESTs to find evidence of their expression. Indeed, 551 (82%) of these clusters showed an overlap with ESTs (432 clusters were fully supported). Only 117 clusters (comprising 775 CSTs) did not show any overlap with known transcribed sequences (14 of these corresponding to pseudogenes according to the human pseudogene database [10]).

**Figure 5:**
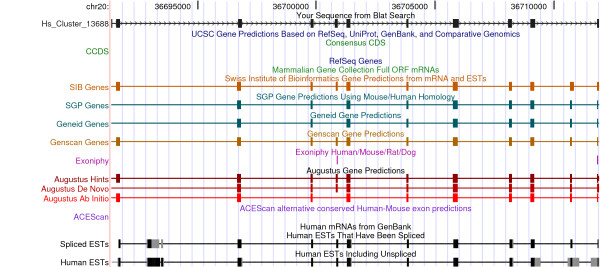
**Cluster n.13688 has been aligned to human genome on UCSC genome browser and compared to annotated genes, gene predictions and ESTs.** The CSTs of this cluster match well to mapped spliced ESTs and exons predicted from various gene-finding programs in a genomic region where no gene has been annotated.

One of the CST clusters possibly corresponding to a novel gene locus is shown in Figure 5. Quite strikingly, coding CSTs not only correspond to spliced ESTs but also to coding exons predicted by a variety of gene finding programs thus further supporting the inferred gene prediction

### Noncoding CSTs

Several evidences have been reported about the critical role of non coding conserved sequences in regulation of gene expression [1] and in particular in the regulation of genes involved in control of development [2].

We are aware that – at this stage – our data are limited to the comparison of human and mouse and may not allow the precise localization of short functional motifs. Nonetheless the identification of core sequence elements shared by several non-coding nrCSTs might represent a powerful approach for the detection of conserved sequences that might be involved in chromatin remodelling or in the regulation of the expression of many genes while unique non-coding nrCSTs might be expected to include elements with more gene-specific functions.

**Figure 6:**
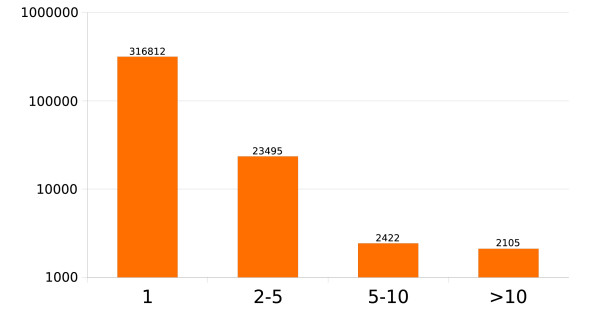
**Abundance of non-coding CSTs in human genome**. Distribution of unique and repeated non-coding CSTs in the human genome. Bars report the number of non-coding CSTs grouped on the basis of their occurrenceData are shown in logarithmic scale.

We performed reciprocal blastn analyses of more than 300,000 human noncoding nrCSTs and observed that nearly 92% of the sequences are unique (ie they do not show any significant similarity with other sequences of the dataset). 7% of sequences show some similarity to up to five different nrCSTs, 1% show up to 10 occurrences while the remaining 0.6% show sequence similarity to more than 10 non-coding nrCSTs. These results are summarized in Figure 6. The observed non-coding repeated elements do not correspond to annotated repeats as the CSTminer analysis was performed on repeat masked sequences.

**Figure 7:**
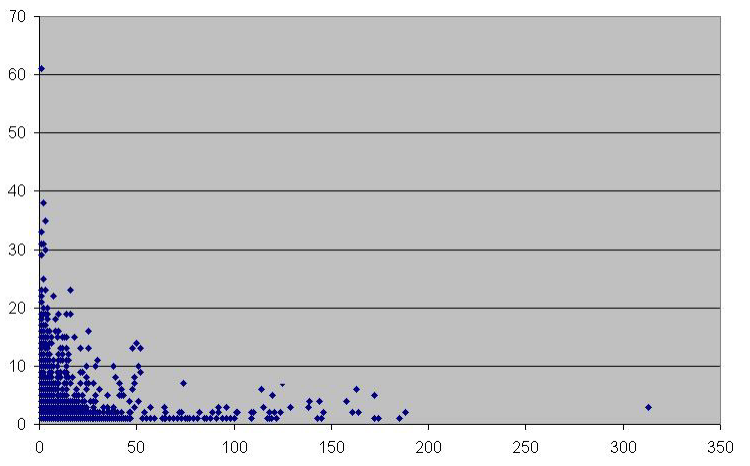
**non-coding CSTs occurrence in mouse and human genome**. Each dot represents the number of occurrences of each non-coding CST in human (x axis) and mouse (y axis) genomes.

Figure 7 shows the frequency of occurrence of sequences corresponding to non-coding nrCSTs in the mouse and human datasets. The plot suggests a remarkable species-specificity of repetitive non coding conserved sequences.

To investigate the hypothesis that noncoding conserved sequences (ncCSTs) might correspond to functional regions we made a comparison with specialized databases containing known regulatory elements. The enrichment of known functional regions in our dataset of conserved non-coding sequences would support the possibility that the same dataset could contain new regulatory elements.

In particular we considered "Presta-promoter", which contains a curated non-redundant set of human promoter sequences [11], Rfam ncRNA database [12] and "OregAnno" database which contains manually curated known regulatory elements [13]. 306 ncCSTs matched with 238 (43%) sequences in the PrestaPromoter dataset, 347 ncCSTs with 269 (21%) "OregoAnno" sequences and 513 ncCSTs with 1069 (3%) Rfam elements. The finding that a sizable proportion of known functional elements are represented in our conserved non-coding set suggests that additional, still unknown, regulatory elements are represented in our ncCST dataset.

### CST comparison with blastz chains

Pre-computed genome alignments are already available for several genomes, including human and mouse and it may make sense to take advantage of this data – provided that information loss is minimal.

We compared CSTs obtained from our full genome analysis with the results obtained by comparing genome regions corresponding to blastz [14] chain tracts only. We adapted the grid based system we developed for the full genome comparison to allow the submission of blastz alignments (or any other "query – target" coordinate pairs) to limit the analysis to these regions. Nonetheless many blastz chains are longer than 100 Kbp (with length up to about 80 Mbp) and cannot be efficiently analyzed with a direct comparison. Those sequences were split in 100 Kbp slices (with 1 Kbp overlap) and – to further reduce computational load – only slices showing at least 3 identical sequences of 10 nt on the same diagonal were compared. This procedure – which is similar to the one employed by the BLAT algorithm [15] – remarkably reduced the number of CSTminer comparisons to about 1% of all the possible 100 Kbp comparisons.

Despite the striking reduction of comparisons we observed that only 1% of total CSTs were completely missed by blastz chains while and additional 4% escaped detection because they did not pass the filter imposing three decamers on the same diagonal above described. However, our data suggest that the use of blastz chains can provide an acceptable reduction of the complexity of analysis with a limited (about 5%) loss of information.

However, the main advantage of blastz chains in this context is their availability as pre-computed features (available for instance at UCSC genome browser website [16]). Indeed, their computation is rather time consuming (481 CPU days for the human – mouse comparison according to [14]).

On the other hand, the reduction procedure based on the identification of exact matches on the same diagonal provides a significant speed up of the process as the computational requirements to perform the identification of those regions is limited (data not shown).

## Discussion

As highlighted in [6] the CSTminer algorithm measures the coding potential through the evaluation of evolutionary dynamics unique to coding sequences not requiring the availability of any annotated feature. Indeed, many analyses of conserved coding or noncoding sequences have been made by classifying a sequence as coding (or non-coding) following a comparison with protein databases [1,17,18]. It is clear that the reliability of such approach depends on the availability of annotated proteins. If a sequence is not supported by a protein it is difficult to decide if the sequence is really noncoding or whether the corresponding protein has simply not been identified.

Moreover very few annotated proteins have been physically sequenced and the vast majority of them are conceptual translation products of available mRNA sequences. This introduces a vicious circle as the hypothetical codingness of a sequence is inferred by the alignment to putative proteins.

We have developed and implemented a high performance grid-based system to perform exhaustive full genome comparisons with CSTminer algorithm to identify and discriminate between conserved coding and noncoding sequences. Besides the speed of the whole procedure even when entire large genomes are compared, one of the main advantages of our system is that it does not require any annotated feature for the assessment of the putative coding potential of identified conserved tracts rendering it useful for the comparison of poorly annotated genomes (i.e. when no or few cDNA sequences are available). It is thus possible to identify "interesting" sequences such as putative genes or regulatory regions, and use these data to drive subsequent experimental analysis or to strengthen the reliability of independent computational data (i.e. de novo gene finding data).

We demonstrated – using as a reference the well annotated human and mouse genomes – that the observation of clusters of coding CSTs is a good indicator of the existence of a gene locus. This information can be incorporated in a gene prediction pipeline where several gene prediction tools are combined and their results compared to limit the rate of false positives and to strengthen the significance of predictions [19].

Collections of conserved non-coding sequences can also address specific studies on sequences that might regulate, for example, gene expression or chromatin structure. Indeed, these data might also facilitate the identification of novel non-coding RNAs, whose importance and prevalence are currently the subject of much debate [20].

Currently available sets of nonding elements are generated following the comparison of the conserved elements with annotated mRNAs. It can thus be expected that following updates of databases some noncoding elements are reclassified as coding. In Table 3 we show a comparison of publicly available conserved noncoding elements with coding CSTs to detect misannotated elements. It is interesting to notice that – as expected – while recent sets of noncoding elements such as CONDOR [21]or Ancora [22] do not show any correspondence to coding CSTs, older sets (reviewed in [23]) show a sizable (239 to 345) number of elements misannotated as noncoding that today we know to be part of coding region of RefSeq sequences.

**Table 3:** Comparison of publicly available noncoding datasets to coding CSTs and currently annotated RefSeq coding sequences.

**Non Coding Elements datasets**	Reference	N° el	CST	Coding CST/RefSeq CDS
**Ancora**	[22]	267641	96%	0
**CONDOR**	[21]	4554	98%	0
Berkeley (*)	[23]	939457	51%	239
Penn State (*)	[23]	452517	85%	261
UCSC (*)	[23]	1007256	59%	345

(N° el:) number of noncoding elements of the dataset; (CST) percentage of elements of the set corresponding to a CST; (Coding CST/RefSeq CDS) number of elements of the set which correspond to a coding CST and to the CDS of a human RefSeq mRNA. (*) Elements of these sets refers to hg10 human assembly. Therefore we preventively converted their coordinates to current assembly by using LiftOver conversion tool.

As pointed out by Couronne [23] local alignment tools, beside the identification of orthologous segments, lead to the identification of paralogous relationships and sequence repetitions. This information is often considered "noise" and is thus removed. This seems reasonable if the primary goal is to align genomes to find large scale orthologous regions; nonetheless repetitive elements can have functional relevance in regulation of gene expression and warrant further inspection. We have used repeat masked sequences – thus purging known repetitive elements – before CSTminer analysis. Nonetheless we observed many highly repetitive conserved noncoding elements that we believe to be interesting and may represent novel lineage specific repetitive elements.

Our analysis system has been implemented on a grid facility [24], taking advantage of the high parallelization achievable and allowing full genome comparisons in very reasonable amount of time (15 days computation to compare mouse and human genomes). Nonetheless many genomes have already been aligned with very sensitive algorithms like blastz and it is possible to take advantage of this information, limiting CSTminer comparison to those regions only.

It is important to notice that although blastz chains are alignments of genomic sequences, the CSTminer alignment step is required to detect local similarities. Indeed the average length of CSTs detected by CSTminer is 190 bases, while blastz tries to extend matches to find large synteny tracts, often resulting in very long alignments.

Use of blastz chains only leads to the complete loss of only about 1% of total CSTs. However, chains longer than 100 Kbp must be split into shorter tracts and an exhaustive comparison of all tracts must be performed. To further improve the speed of the analysis it is possible to limit CSTminer comparison of chain fragments to pairs that show at least 3 identical matches of 10 nt on the same diagonal. By applying this restriction it is possible to drastically limit the number of comparisons (nearly 1% of total comparisons) thus reducing the computation time to slightly more than 24 hours (in the case of human and mouse genomes) with a further loss of 4% of CSTs.

## Conclusion

In this paper we describe a grid-based system devised to perform full genome comparisons with CSTminer algorithm. The main advantage of this system is that the assessment of coding potential of conserved sequences does not require any annotated feature rendering it useful for the comparative analysis of poorly annotated genomes.

The system has been benchmarked on the well-annotated human and mouse genomes where it proved its reliability.

## Methods

### CSTminer

The CSTminer algorithm has been described in [6] and slightly modified in [7] where a web interface to run CSTminer was made available. A further automatic web system to compare a single sequence to several genomes has also been implemented in [25].

Briefly, given a pair of sequences, CSTminer identifies high scoring segment pairs (HSPs) through a Blast-like sequence comparison. The coding capacity of each CST delimited by an HSP is then assessed by assigning a coding potential score (CPS) which corresponds to the maximum score value obtained from each of the possible reading frames in the forward and reverse orientation.

CSTminer also allows the display of the highest-scoring triplet window (default minimum length of 60 nt) by scanning each detected CST. This approach facilitates the detection of potential coding regions located in longer CSTs which might contain both coding and non-coding tracts (through the presence of untranslated mRNA or intronic regions).

Following an accurate benchmark on controlled coding and non-coding datasets, CPS thresholds for coding and non-coding CSTs were evaluated. Therefore each CST was labeled as coding (**Cod) **(if CPS ≥ coding_threshold) or non-coding (**NonCod) **(if CPS ≤ non_coding threshold or CPS < coding_threshold and highest scoring triplet window CPS < coding threshold). CSTs with CPS non fulfilling these requirements were labeled as **Undefined**. Finally CSTs with more than 95% of similarity were labelled as **Ultrancoserved **and no CPS was computed as the low divergence would not allow the computation of a significant score.

### GRID

We empirically determined that for large scale comparisons CSTminer gives optimal results with sequences of 100 Kbp with 1 Kbp overlap. Indeed this value allows a good balance between computational speed and the occurrence of CST fragmentation at the border of the submitted sequences.

In order to reduce the time needed to execute this large amount of comparisons, we took advantage of grid technology using many machines in parallel. Indeed each CST comparison requires an independent computation thus we split all the 800 M comparisons in smaller subset and we run them on the EGEE grid infrastructure [24].

In order to maximize the level of parallelization, the comparisons were grouped in set of 1000 (10 human 100 K slices vs. 100 mouse 100 K slices). The number of comparisons was chosen in order to have each task running for approximately one hour, giving a good ratio between the time spent in order to set-up the environment and the CPU time spent in running the comparison. This approach also assures something similar to a check-point: even if a job fails only less than one hour of computation is lost.

### Clustering

The clustering procedure is an improvement of the procedure described in [8]. The basic idea is to identify genomic regions with a significant concentration of coding CSTs.

Given N coding CSTs sorted on their genomic start we computed *t*_3*i*_, the genomic span of three consecutive CSTs centered in CST i for *i *∈ (2, *N *- 1). We labeled as pre-cluster three consecutive coding CSTs centered in CST i if 2 * t_3i _≤ $\bar{t_{\text{3,60}}}$ where $\bar{t_{\text{3,60}}}$ is the average genomic span of *t*_3k _for *k *∈ (*i *- 30, *i *+ 30). When i<30 or i>N-30, $\bar{t_{\text{3k}}}$ is computed respectively for *k *∈ (2,60) or *k *∈ (*N *- 61, *N *- 1). Overlapping pre-clusters are then merged into clusters.

The main difference with the previous clustering procedure is that clustering parameters (average density of surrounding CSTs) are now dynamically computed over a 60 CSTs window in the genomic region under analysis – thus accounting for regions with different gene density.

Given that each CST has human and mouse genomic coordinates, the clustering procedure is applied both to human and mouse genomes. Only CSTs belonging to a cluster in both organisms are considered. As already pointed out clusters are computed on coding CSTs only, but syntenic CSTs of all classes (non-coding, undefined and ultraconserved) are included in clusters following a post-processing step.

## Authors' contributions

FM carried out the analysis on coding dataset, participated in the design and coordination of the study and helped to draft the manuscript. AA carried out the analysis on non-coding dataset, GG adapted the software to the grid-based application. GD and GPM set up and run the grid-based procedure. GP conceived the study and helped to draft the manuscript. All the authors read and approved the final manuscript.
